# Efficacy of Wholetones^®^ 2Sleep and classical music on sleep and health behaviors of adults with insomnia symptoms: A single blind, randomized, controlled, crossover pilot trial

**DOI:** 10.5935/1984-0063.20190091

**Published:** 2019

**Authors:** Heather Hausenblas, Stephanie Hooper, David Hooper, Kevin Coyle, Tarah Lynch

**Affiliations:** 1 Jacksonville University, Kineisology - Jacksonville - FL - United States.

**Keywords:** Sleep Disorders, Music, Anxiety, Fatigue, Mood

## Abstract

**Objectives:**

To conduct a randomized single-blind placebo-controlled crossover trial on adults with insomnia symptoms to examine the efficacy of Wholetones^®^ 2Sleep Music (WM) and Classical Music (CM) on sleep quality, anxiety/stress, fatigue, productivity, and mood.

**Methods:**

Following baseline assessments, 38 adults (M age = 46.6 years) were randomized to either WM or CM conditions for 10 days and then the alternative music for 10 days after a 4 day “wash-out”. The outcomes were sleep quality (i.e., Pittsburgh Sleep Quality Index) and psychological measures.

**Results:**

Listening to both the WM and CM resulted in significant improvements from baseline for sleep quality, stress/anxiety, fatigue, productivity, and mood (*p*<0.05).

**Conclusion:**

WM and CM provides a simple, noninvasive, and non-pharmacological intervention to promote sleep quality resulting in improved daytime mood, fatigue, productivity, and anxiety/stress.

## INTRODUCTION

Insomnia is the most common sleep disorder, and it is characterized by persistent difficulties either initiating or maintaining healthy sleep behaviors and daytime impairments^1^. Representing a major health problem insomnia is associated with decreased quality of life, mood, mental health (e.g., anxiety, depression), and functional and cognitive abilities^2^. Between 33 to 50% of adults experience insomnia symptoms^3^^,^^4^. Common interventions to improve insomnia symptoms include over-the-counter and prescribed drugs; which are criticized for their side effects, short- and long-term efficacy, and dependency.

Music may provide a healthier, safer, and natural alternative to over-the-counter and prescription sleep aids to improve sleep quality and overall health^5^^,^^6^. For example, a recent meta-analysis found that listening to music prior to nighttime sleep resulted in improved sleep (as determined by the Pittsburgh Sleep Quality Index) compared to usual care^7^. Most of the research examining the effects of music on sleep has been conducted in clinical populations (e.g., chronic insomniacs, hospital patients^8^); and thus a need exists to examine the effects of music on sleep quality for people with transient insomnia^9^.

Limitation of previous research examining the effects of music on sleep include methodological issues such as baseline and between group differences, lack of assessments in the home environment, and lack of assessments on the daytime consequences of the intervention^6^^,^^10^^-^12. In short, music listening at bedtime appears to have a positive impact on sleep quality, but further research is needed to determine its effects on nonclinical populations, in particular in the home environment, as well as its daytime consequences using crossover designs.

Of importance, a limited number of studies have directly compared the effects of different types of music. Typical study designs have compared a music condition to other nonmusic conditions [e.g., audiobooks or waitlist controls^6^]. Thus, a need exists to compare different types of music to determine the moderating effect of music genre on sleep outcomes as well as daytime mood and productivity. Prior research suggests that listening to classical musical prior to nighttime sleep, compared to other genres (e.g., meditation, New Age), may be the most beneficial for improving quality of life, anxiety levels, and cardiovascular outcomes^13^^-^15. As well, classical music is the most popular music genre listened to by people prior to sleep^9^. Trahan et al.^9^ suggested that a next logical step for future research is to compare different music classes to better understand their effectiveness and determine which type of music best supports improved sleep in real world situations.

To address these research shortcomings the purpose of this trial was to examine the efficacy of different types of music (i.e., Wholetones^®^ 2Sleep [WM] and Classical Music [CM]) to improve sleep quality as well as the daytime consequences of the intervention in adults with insomnia symptoms in their home environment. Wholetones^®^ 2Sleep is music that is designed to lull the listener into a deep, delta sleep, using frequency-enhanced music and precise tempos.

The primary outcome was sleep quality as assessed by the Pittsburgh Sleep Quality Index. Secondary outcomes included daytime fatigue, mood, perceived stress, anxiety, and productivity. We hypothesized that both music conditions would result in improved sleep and self-reported health outcomes, with larger positive effects evidenced for the WC compared to the CM.

## METHODS

### Participants

**Figure 1:**
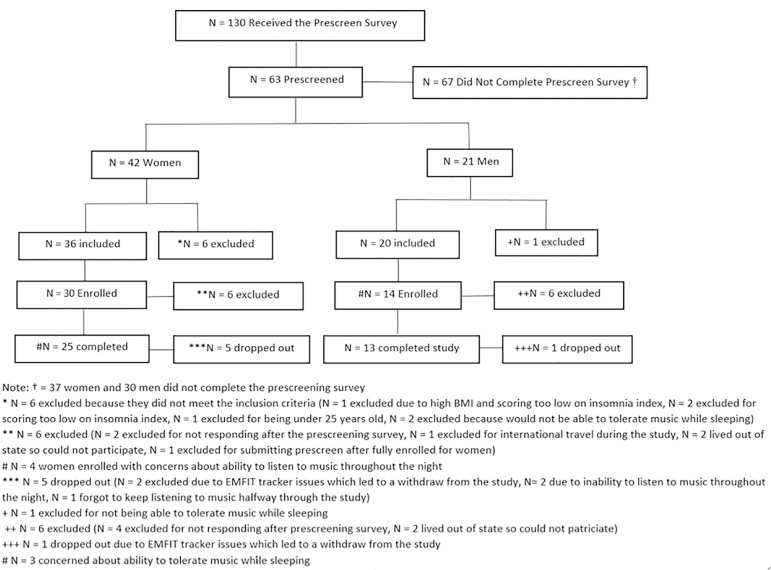
Flow chart of the participant recruitment.

Participants included 38 adults (*n*=25 women and *n*=13 men; age range = 29 - 64 years; *M* age = 46.6 years, *SD* = 9.04) who reported experiencing insomnia symptoms (as determined by the Insomnia Severity Index; Bastien et al., 2001). Individuals were excluded if they smoked, were either pregnant or trying to conceive, diagnosed with a sleep disorder, were taking sleep supplements or medication, or had a BMI greater than 32 (see Figure 1 for the Participant Flow Chart).

### Procedures and Design

This study received IRB approval. Using a single-blind crossover design, following completion of one week of baseline assessments the participants were randomized to either the WM or CM condition for 10 days. Following a 4 day “washout period” the participants listened to the alternative music condition for 10 days. The participants were blinded to the music condition and they were instructed to listen to the music for 30 minutes prior to bed. The music would continue to play throughout the night unless the participants turned it off.

Participants completed a daily diary assessing sleep quality, music likeability, and adherence. They also completed self-report assessments of their mood, daytime fatigue, anxiety, perceived stress, productivity, and sleep quality at baseline and on day 10 of each music condition. Continuation of baseline lifestyle behaviors were encouraged for the study duration. A total of 44 adults were enrolled and 38 participants completed the trial, representing an adherence rate of 86.36%. Reasons for dropout included difficulties listening to the music or inability to complete the assessments.

### Music Intervention

The CM playlist was selected based on the Mayo Clinic and NIH music recommendations for better sleep that consisted of Beethoven (i.e., Moonlight Sonata, first movement), Marconi Union (i.e., Weightless), Chopin (i.e., Nocturne No.2, Op.9), Ravel (i.e., Piano Concerto in G major, 2nd movement), and J.S. Bach (i.e., Prelude No.1). The WM is unique in that it employs a proprietary method of tuning and layering the music with a specific frequency underlayment.

### Measures

The following psychometrically validated self-report measures were completed at day 0 and day 10 of each music condition: Pittsburgh Sleep Quality Index (PSQI) which assesses sleep quality and patterns^16^; Profile of Mood States (POMS) which assesses the mood states of tension, anger, vigor, fatigue, depression, and confusion^17^; Flinders Fatigue Scale that measures various characteristics of daytime fatigue^18^; Trait Anxiety Inventory which assesses anxiety levels^19^; Perceived Stress Scale which measures the degree to which situations are appraised as stressful^20^; and daytime productivity was assessed with a single item assessing satisfaction with ability to complete daily activities [from the Life Satisfaction Scale^21^].

A daily diary was also completed which assessed adherence, music likeability, sleep quality, and any issues with listening to the music. Music enjoyment was assessed on a 10-point Likert scale with 1 representing very unenjoyable and 10 very enjoyable. Sleep quality with music was assessed on a 10-point Likert scale with 1 representing less sleep quality improvement and 10 the most sleep quality improvement. Falling asleep more easily with music was assessed using a 10-point Likert scale with 1 representing not helpful settling down and 10 very helpful for settling down. Feeling rested after listening to music was assessed on a 10-point Likert scale with 1 representing less rested/more tired and 10 feeling most rested.

### Data Analysis

The data were analyzed with SPSS. Paired sample *t*-tests were used to analyze mean differences between groups using delta scores. Chi-squared analysis was used to examine differences in number of individuals who improved with each condition from baseline. 2 (Condition: Wholetones *vs.* Classical Music) x 2 (Time: Baseline *vs.* Music Condition) repeated measures analysis of variance (ANOVAs) were used to examine condition, time, and interaction effects. The significance level was set at *p*≤0.05.

## RESULTS

Regarding recruitment, 130 individuals expressed interest in the study and received the prescreen assessment, of which 63 completed the prescreen assessment (*N*=42 women and *N*=21 men). Nineteen of the 63 prescreened individuals were excluded for not meeting inclusion criteria, leaving enrollment at 44. Of the 44 enrolled participants, 6 dropped out due to difficulty listening to the music or poor adherence with listening to the music. A total of 38 subjects complete the study (*N*=25 women and *N*=13 men; Figure 1).

The participants baseline Insomnia Severity Index Scores reflected subthreshold to moderate insomnia at baseline (range=7 to 21), with a mean score of 11.00 (*SD*=4.67). Regarding baseline insomnia issues 71.05% (*n*=27) had difficulties falling asleep, 89.47% (*n*=34) had difficulty staying asleep, and 81.58% (*n*=31) woke up too early.

### Primary Outcome (PSQI)

A significant main effect for time revealed that the participants had significant improvements in their PSQI scores following both music conditions. No significant condition or condition x time interactions were evidenced (Table 1). Regarding categorical sleep quality, a total score of less than “5” on the PSQI is indicative of good sleep quality. At baseline 18.41% (*n*=7) of the participants were classified as good sleeper. Significantly more participants were classified as good sleepers following listening to WM (57.89%, *n*=22, χ^2^=12.55, *p*<.01) and CM (52.63%, *n*=20, χ^2^=9.71, *p*<.01) compared to baseline.

**Table 1:** Statistics for the profile of mood states, anxiety, fatigue, productivity, and perceived stress measures.

Outcome	Baseline	Wholetones Music	Classical Music	Main effect time	Main effect condition	Interaction
	Mean (SD)	Mean (SD)	Mean (SD)	F(1,74)	F(1,74)	F (1,74)
*Trait Anxiety	36.39 (10.06)	33.55 (9.84)	35.24 (8.75)	4.66, *p*=0.03	0.68, *p*=0.41	0.18, *p*=0.67
*Perceived Stress	13.34 (7.36)	10.39 (6.63)	10.45 (6.37)	19.89, *p*<0.01	0.00, *p*=0.97	0.00, *p*=0.99
*POMS Total	52.47 (29.28)	38.26 (20.51)	43.13 (21.82)	16.28, *p*<0.01	0.64, *p*=0.43	0.24, *p*=0.63
*POMS Depression	6.34 (7.49)	3.34 (4.67)	4.11 (5.58)	13.27, *p*<0.01	0.27, *p*=0.61	0.09, *p*=0.77
*POMS Anger	6.76 (7.20)	3.71 (4.29)	4.32 (4.54)	13.37, *p*<0.01	0.16, *p*=0.69	0.70, *p*=0.79
POMS Vigor	15.95 (6.62)	15.18 (6.35)	16.53 (6.17)	0.02, *p*=0.88	0.85, *p*=0.36	0.27, *p*=0.60
*POMS Confusion	5.89 (3.83)	4.63 (3.57)	5.47 (3.45)	5.55, *p*=0.02	1.30, *p*=0.26	0.31, *p*=0.58
*POMS Tension	8.76 (5.61)	5.68 (4.37)	6.11 (3.77)	26.76, *p*<0.01	0.14, *p*=0.71	0.05, *p*=0.83
*POMS Fatigue	8.76 (4.75)	5.71 (3.45)	6.61 (4.23)	28.42, *p*<0.01	0.79, *p*=0.38	0.27, *p*=0.60
*PSQI Global	6.47 (2.77)	4.52 (2.00)	4.73 (2.04)	39.36, *p*<0.01	0.13, *p*=0.72	0.05, *p*=0.82
*Productivity	3.00 (0.66)	3.16 (0.72)	3.16 (0.64)	18.97, *p*<0.01	0.00, *p*=0.98	0.00, *p*=0.99
*Flinders Fatigue	11.08 (5.87)	8.60 (5.46)	8.63 (4.83)	4.66, *p*=0.03	0.00, *p*=1.00	0.00, *p*=1.00

POMS=Profile of Mood States; PSQI=Pittsburgh Sleep Quality Index

* significant main effect for time, *p*<.05

### Secondary Outcomes

Significant main effects for time revealed that the participants had significant improvements in their daytime fatigue, POMS, Perceived Stress, Anxiety, and Productivity following both music conditions. No significant condition or condition x time interactions were evidenced (Table 1).

### Adherence, Likeability, and Adverse Events

Both music conditions had an adherence rate of ~85% for listening to the music 30 minutes prior to sleep. Both conditions reported similar responses to items regarding enjoyment of the music, sleep quality with music, finding it easier to fall asleep with the music, and feeling more rested in the morning after listening to the music indicating that both types of music were equally likeable and enjoyable (Table 2). No issues were reported by the participants for listening to the music.

**Table 2:** Perception of the music by condition.

	Wholetones Mean (S/D)	Classical Mean (S/D)	Statistic
Was the music enjoyable?	4.63 (3.17)	4.10 (3.02)	*t*=0.740, *p*=0.461
Sleep quality compared to no music	4.44 (2.81)	3.86 (2.30)	*t*=0.983, *p*=0.329
Easier to fall asleep with music?	4.94 (3.14)	4.41 (2.95)	*t*=0.751, *p*=0.455
Feeling rested in morning after music	4.89 (2.41)	4.52 (2.62)	*t*=0.637, *p*=0.526

No significant group differences evidenced.

## DISCUSSION

The purpose of this trial was to examine the efficacy of different types of music (i.e., WM and CM) to improve sleep quality as well as the daytime consequences of the intervention in adults with insomnia symptoms. Consistent with our hypothesis, we found that both music conditions had improved sleep quality and self-reported health outcomes. In contrast to our hypothesis, we found that both music conditions were equally effective in improving sleep quality and daytime mood, fatigue, and perceived stress. In short, our findings revealed that listening to either CM or WM prior to nighttime sleep in the home environment resulted in significant improvements in sleep quality, daytime mood, productivity, fatigue, perceived stress, and anxiety levels. These findings are consistent with research revealing that listening to music resulted in reduced insomnia symptoms in clinical populations as well as in hospital settings^12^.

Our results illustrate that listening to music results in improved sleep quality and daytime mood in nonclinical populations (i.e., do not have a sleep disorder but have insomnia symptoms) in the home environment. Although pharmaceutical and over-the-counter sleep aids may provide some relief for sleep issues, many are ineffective and can lead to short term and chronic health-related side effects. Music’s potential to improve sleep quality as well as daytime mood and productivity makes it a viable, low cost, side-effect free option for potentially improving sleep quality.

Strengths of our study include the crossover design in the home environment, comparison of two types of music, standardized self-report measures, and assessment of the daytime consequences of the music intervention. Limitations of our study include a lack of a nonmusic control group and objective sleep assessments.

Future researcher should examine how music longitudinally impacts the physiology and pathways associated with sleep in a variety of populations and environments. In summary, the WM and CM were reported as moderately likeable and enjoyable and represent a simple, noninvasive, and non-pharmacological intervention to promote improved sleep quality/quantity, mood, anxiety, fatigue, and stress with adults who experience insomnia symptoms.
